# A new Carboniferous edaphosaurid and the origin of herbivory in mammal forerunners

**DOI:** 10.1038/s41598-023-30626-8

**Published:** 2023-04-05

**Authors:** Arjan Mann, Amy C. Henrici, Hans-Dieter Sues, Stephanie E. Pierce

**Affiliations:** 1 Department of Paleobiology, National Museum of Natural History, Smithsonian Institution, MRC 121, P.O. Box 37012, Washington, DC 20013-7012 USA; 2 Section of Vertebrate Paleontology, Carnegie Museum of Natural History, 4400 Forbes Avenue, Pittsburgh, PA 15213 USA; 3 Museum of Comparative Zoology and Department of Organismic and Evolutionary Biology, Harvard University, Cambridge, MA 02138 USA

**Keywords:** Evolution, Palaeontology, Phylogenetics, Taxonomy

## Abstract

Herbivory evolved independently in several tetrapod lineages during the Late Carboniferous and became more widespread throughout the Permian Period, eventually leading to the basic structure of modern terrestrial ecosystems. Here we report a new taxon of edaphosaurid synapsid based on two fossils recovered from the Moscovian-age cannel coal of Linton, Ohio, which we interpret as an omnivore–low-fibre herbivore. *Melanedaphodon hovaneci* gen. et sp. nov. provides the earliest record of an edaphosaurid to date and is one of the oldest known synapsids. Using high-resolution X-ray micro-computed tomography, we provide a comprehensive description of the new taxon that reveals similarities between Late Carboniferous and early Permian (Cisuralian) members of Edaphosauridae. The presence of large bulbous, cusped, marginal teeth alongside a moderately-developed palatal battery, distinguishes *Melanedaphodon* from all other known species of Edaphosauridae and suggests adaptations for processing tough plant material already appeared among the earliest synapsids. Furthermore, we propose that durophagy may have provided an early pathway to exploit plant resources in terrestrial ecosystems.

## Introduction

The origin of herbivory in amniotes is intimately linked with the origin of modern terrestrial ecosystem structure and an expansion of niche exploitation^1–3^. Currently, it is thought that the ability to efficiently process plant material was well-established by the early Permian, with groups including diadectid stem-amniotes, captorhinid and bolosaurid reptiles, as well as caseid and edaphosaurid synapsids being the first tetrapod lineages to adopt this feeding strategy^4,5^. Whereas most early Permian members of the two synapsid clades are interpreted as high-fibre herbivores, such as the iconic large-bodied *Cotylorhynchus* (Caseidae) and *Edaphosaurus* (Edaphosauridae), virtually all Late Carboniferous representatives of these synapsid lineages are smaller faunivorous forms that likely preyed on insects or other small animals^6–8^. Therefore, there is an apparent lack of transitional Carboniferous synapsids with less-specialized dietary preferences such as omnivory and low-fibre herbivory, which would bridge the gap between ancestral carnivores/insectivores and high-fibre herbivores characteristic of the Permian^3,5^ (Hotton et al., 1997; Reisz and Sues, 2000). Part of this gap may be explained by the still insufficiently documented Late Carboniferous fossil record of early amniotes, which has recently started to reveal unexpected morphological and ecological diversity (e.g., ^9,10^).

Recently, a series of papers^11,12^ have used various modelling approaches to address the origins of herbivory in tetrapods, ultimately predicting its appearance during the mid-Carboniferous shortly after the origin of amniotes. While quantitative approaches can be powerful, confirmation of the resulting hypotheses can only be established through the discovery of new fossil material. Here we provide such a new record, reporting a new species of edaphosaurid synapsid from the famous Late Carboniferous (Pennsylvanian: Moscovian) fossil locality of Linton, Ohio. The vertebrate-bearing cannel coal from Linton, Ohio, likely represents an abandoned channel or oxbow lake that was an allochthonous deposit of sapropelic plant material^13^. Although this deposit is well known for being particularly rich in fish and amphibian remains^14^, terrestrial faunal components such as amniotes are rarely found (e.g. ^9,10,15^). The new edaphosaurid material is preserved on two blocks of cannel coal that collectively document a good portion of the skull, including the distinctive marginal dentition. Its dentition reveals features indicative of omnivory or low-fibre herbivory, placing this animal among the earliest known tetrapod herbivores and certainly the oldest known synapsid herbivore. This new discovery also offers additional insights into the early evolution of herbivory among tetrapods and their ecosystems, revealing the importance of durophagous specialists in establishing these early guilds. Furthermore, the presence of this taxon in the Middle Pennsylvanian offers the earliest known fossil evidence of divergent feeding strategies and niche expansion among amniotes, which occurred during the ‘wet-phase’ or coal swamp environments of the mid-Late Carboniferous.

## Methodology

Specimens used in this study are housed at the following institutions: American Museum of Natural History (AMNH), New York, New York; Carnegie Museum of Natural History (CM), Pittsburgh, Pennsylvania; Field Museum of Natural History (FMNH), Chicago, Illinois; Museum of Comparative Zoology at Harvard University (MCZ), Cambridge, Massachusetts; and USNM, National Museum of Natural History, Washington, District of Columbia. Fossils were photographed with a Canon EOS 6 with a Canon Macro EF 100 mm lens. Digital photographs were processed and figures were assembled using Adobe Illustrator CS6. CM 93778 and CM 93779 were microCT–scanned and digital peels were generated and rendered into stereolithography files at the University of Texas at Austin CT scanning facility. Stereolithography files of the digital peels were 3D printed using a Stratasys Connex500 Polyjet printer for observation. Processing and printing were conducted by 3DPhacktory in Toronto, Ontario, Canada.

### Systematic palaeontology

- Synapsida Osborn^16^.
- Sphenacomorpha Ivakhnenko^17^ *sensu* Spindler et al.^18^.
- Edaphosauridae Cope^19^.
- *Melanedaphodon hovaneci* gen. et sp. nov.

#### Holotype

**Figure 1:**
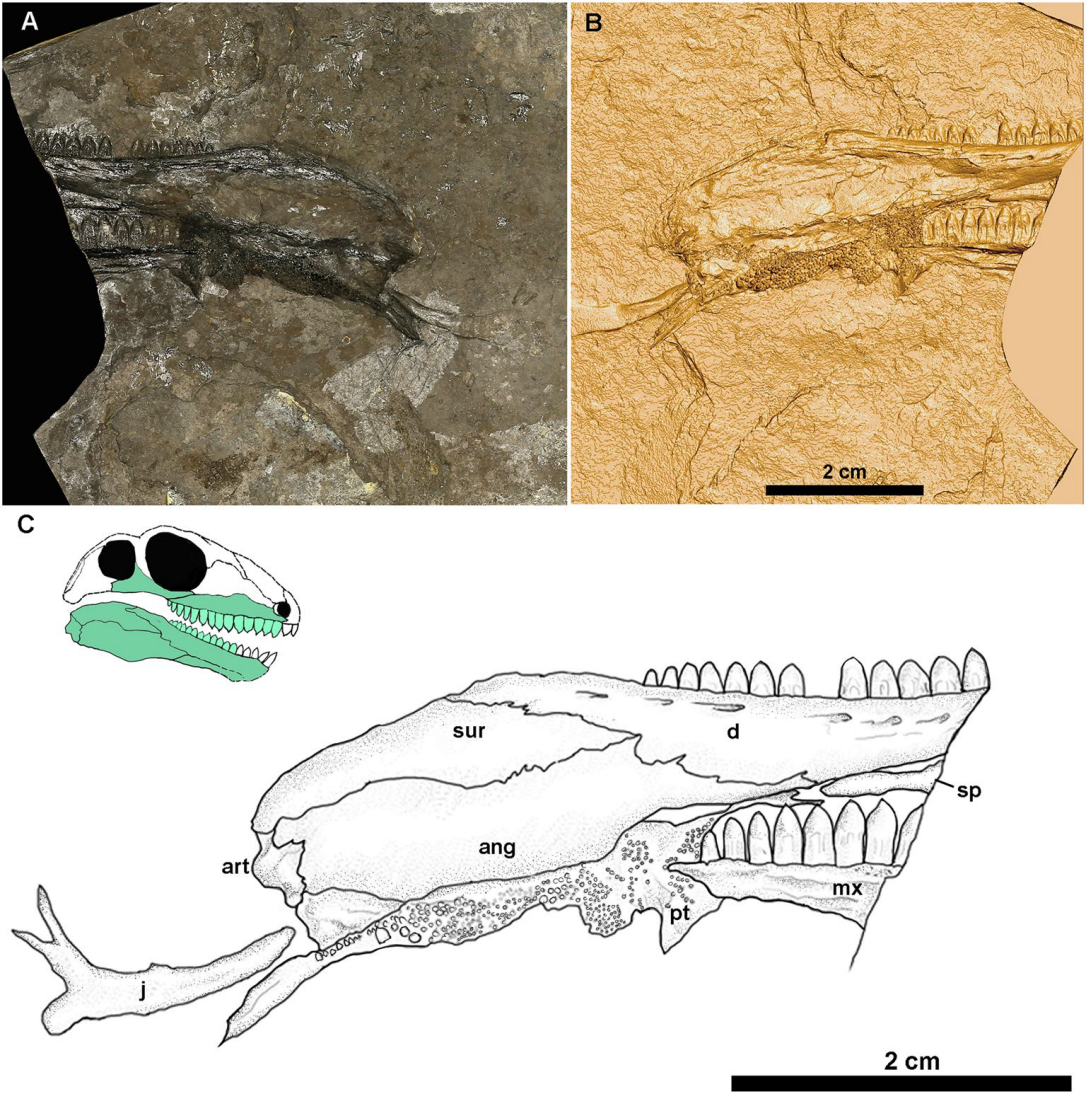
Holotype of *Melanedaphodon hovaneci* gen. et sp. nov., CM 93778. (**A**) Photograph showing the negative relief/natural mould. (**B**) Digital three-dimensional rendering of the CT data in positive relief. (**C**) Interpretative drawing of specimen based on micro-CT data and original fossil. Top left corner shows a reconstruction of the skull in lateral view with the preserved cranial elements highlighted. Anatomical abbreviations: *ang* angular, *art* articular, *d* dentary, *j* jugal, *mx* maxilla, *pt* pterygoid, *sur* surangular, *sp* splenial.

CM 93778, a natural mould of cranial remains comprising a partial right mandibular ramus, a right pterygoid, the posterior part of a right maxilla, and the right jugal (Fig. 1). Donated to CM by Scott McKenzie.

#### Referred material

**Figure 2:**
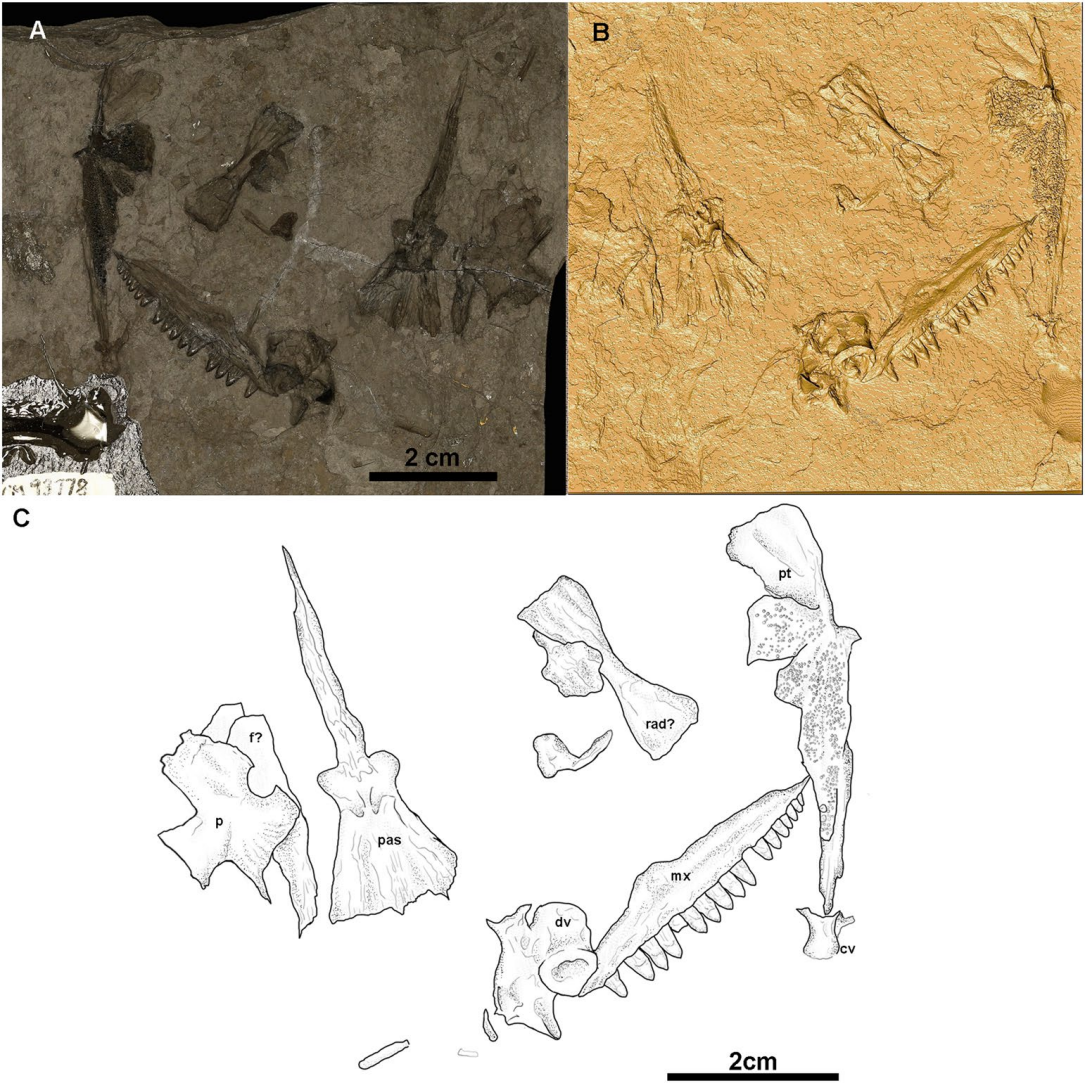
*Melanedaphodon* hovaneci gen. et sp. nov., CM 93779. (**A**) Photograph showing the negative relief/natural mold. (**B**) Digital three-dimensional rendering of the CT data in positive relief. (**C**) Interpretative drawing based on the CT scans and original fossil. Anatomical abbreviations: *cv* caudal vertebra, *f* frontal, *mx* maxilla, *p* parietal, *pas* parabasisphenoid, *pt* pterygoid, *rad* radius, *dv* dorsal vertebra.

CM 93779, a natural mold of partial skeleton comprising a right maxilla, a right pterygoid, a left parietal, a left frontal, a parabasisphenoid, and postcranial bones (Fig. 2). Collected by John Spina and donated by Scott McKenzie.

#### Locality and horizon

Coal mine operated originally by the Ohio Diamond Coal Company at Linton in Saline Township, Jefferson County, Ohio, U.S.A. (for details see Hook and Baird, 1986). Local cannel coal immediately below the Upper Freeport coal, Allegheny Group. Middle Pennsylvanian (Moscovian).

#### Etymology

Generic name derived from the combination of the Greek ‘*melanos*’ meaning ‘black’ and ‘*edaphon*’ meaning ‘pavement’ and ‘*odon*’ meaning ‘tooth’, referring to the dense shagreen on the pterygoid and to the position of the taxon among Edaphosauridae. The specific epithet *hovaneci* honors George Hovanec who generously donated funds to facilitate the CT scanning of Linton fossils.

#### Diagnosis

An edaphosaurid synapsid with the following autapomorphies: long maxilla with 20 tooth positions; marginal dentition consisting of tall teeth with bulbous crowns that have pointed apices; and cutting edges of tooth crowns without serrations. Further differential diagnosis includes: an elongate pterygoid shared with *Ianthasaurus* but not *Edaphosaurus*. Palatal shagreen with enlarged teeth on the anterior (palatal) ramus of pterygoid shared with *Ianthasaurus* but not *Edaphosaurus.* Differs from *Ianthasaurus *but shares with *Edaphosaurus* in having a tooth battery instead of enlarged single tooth row on the transverse flange of the pterygoid. Differs from *Gordodon* in the absence of a diastema on the anterior end of the maxilla.

#### Comments

Spindler et al.^20^ briefly redescribed the putative bolosaurid “*Belebey” augustodunensis* from the early Permian (Artinskian) of France^21^ and reinterpreted it as a probable edaphosaurid. This reassessment was based on the peculiar bulbous teeth of the holotype, which lacks the diagnostic ‘offset heel’ or ‘shelf’ on the teeth of bolosaurid parareptiles such as *Belebey* and *Bolosaurus*. Spindler et al.^20^ considered “*Belebey” augustodunensis* a nomen dubium. We concur with their assessment and find a close similarity between the teeth of this taxon and those of the new edaphosaurid *Melanedaphodon hovaneci*. Both appear to possess distinct bulbous teeth with cutting edges on the crowns. Although it is likely that “*Belebey” augustodunensis* is an edaphosaurid, perhaps closely related to *Melanedaphodon*, additional material is needed in order to confirm this reassignment.

### Statement of permission for study

All new fossil specimens reported in this paper (CM 93778 and CM 93779) are permanently housed at the Carnegie Museum of Natural History. All specimens were studied with permission from the Carnegie Museum of Natural History collections and curatorial staff (Matthew C. Lamanna).

## Description

The available material of *Melanedaphodon hovaneci* consists of skeletal remains of two individuals preserved on separate blocks of cannel coal, CM 93778 and CM 93779 (Figs. 1, 2). These specimens both represent individuals at perhaps slightly different growth stages, as evident from the different dimensions of the maxilla, with CM 93779 being slightly smaller. The holotype, CM 93778, preserves most of the right mandibular ramus in lateral perspective, including the posterior portion of the dentary, the anterior part of the splenial, and the angular, surangular, and articular (Fig. 1). There is also a well-preserved, disarticulated right pterygoid preserved in ventrolateral aspect, the posterior part of the right maxilla with teeth in medial view, and an almost complete right jugal in lateral aspect. CM 93779 presents a nearly complete right maxilla in lateral aspect, the ventral aspect of a crushed left pterygoid, a well-preserved left parietal in dorsal aspect, a probable left frontal, and the ventral surface of the parabasisphenoid (Fig. 1). The postcranial skeleton of CM 93779 is represented by a radius, a caudal vertebra, and an incomplete dorsal vertebra (lacking most of the neural spine). Descriptions of the elements below are based on both specimens.

**Figure 3:**
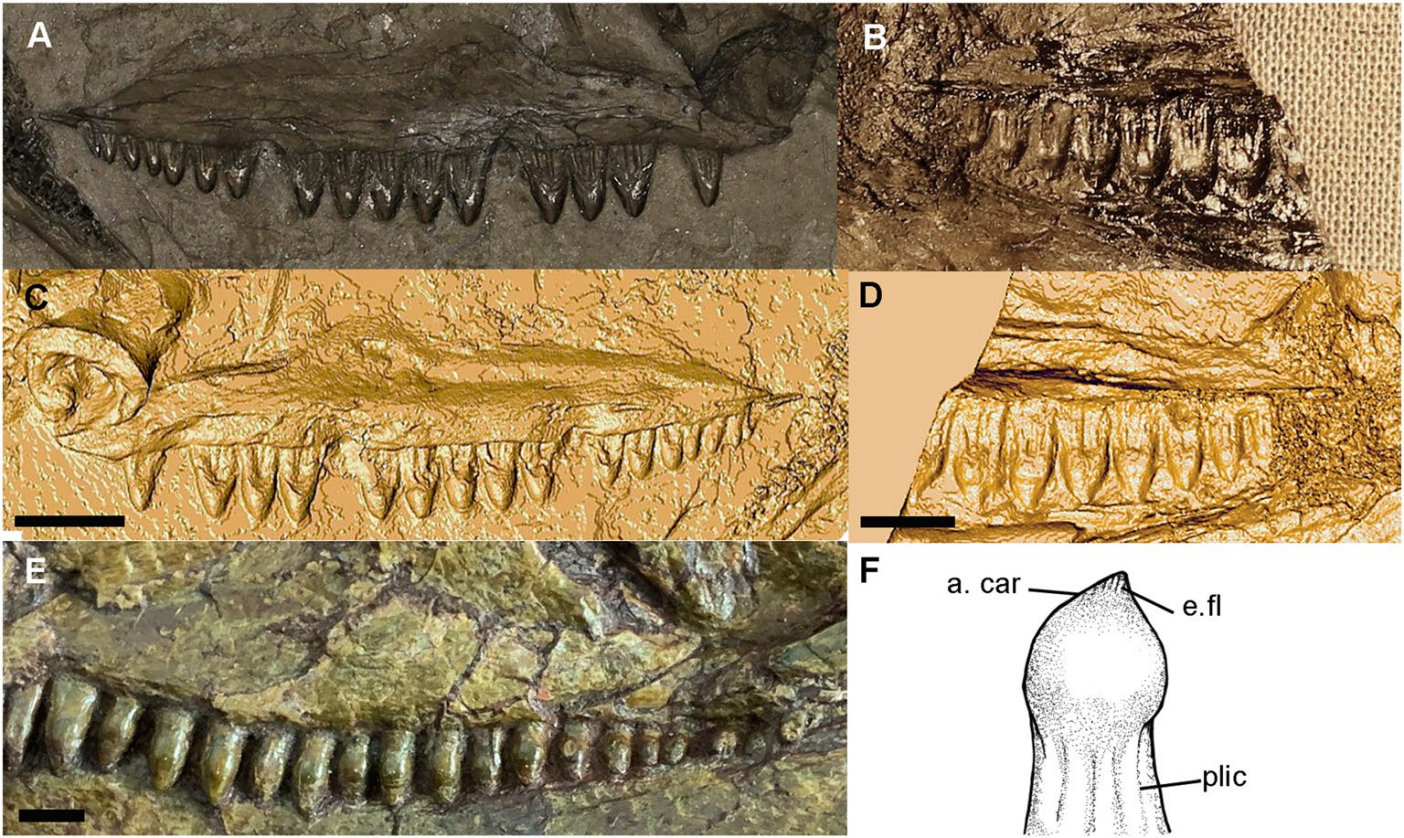
(**A**, **C**) Photograph and digital three-dimensional rendering of the maxilla of CM 93779. Digital render shows the positive relief and true anatomy of CM 93779 revealing 16 wide-based teeth with bulbous tips. (**B**, **D**) Photograph and digital three-dimensional rendering of the maxilla of CM 93778. Digital render shows the positive relief and true anatomy of the medial surface of the maxilla on CM 93778, revealing the bulbous tooth tips from a different perspective. (**E**) Maxilla of a specimen of *Edaphosaurus* sp. (USNM PAL 299844), showing teeth with bulbous crowns with nozzle-like apices. (**F**) Reconstruction of a tooth of *Melanedaphodon hovaneci* gen. et sp. nov., highlighting the bulbous crown. Anatomical abbreviations: *a.car* apical carina, *e.fl* enamel fluting, *plic* plicidentine.

The maxilla of *Melanedaphodon* is long with a moderately developed facial lamina that rises midway along the maxilla at the level of the largest teeth in the tooth row (Figs. 1, 2). It has a short posterior process. Anteriorly, a small subnarial process likely contributed to the ventral margin of the external narial opening. The lateral surface of the maxilla is slightly rugose and pierced by small neurovascular foramina. The more complete maxilla has spaces for approximately 20 teeth (Fig. 3).

The teeth of *Melanedaphodon* are distinctive in possessing bulbous tooth crowns with apical cutting edges and tall roots. Proportional to the size of the maxilla, the teeth of *Melanedaphodon* appear much broader than those of other known edaphosaurids. The exposed portion of the tooth bases or necks reveal a pattern of infolding that indicates the presence of plicidentine^9^. The shape of the teeth is similar to that seen in *Edaphosaurus*, but the crowns are more expanded and have well-defined apical carinae. Although there may be some variation along the tooth row, some of the larger teeth on CM 93778 do not show clearly defined cutting edges but instead appear to terminate in an offset apex, giving the appearance of a nozzle-like tip as in *Edaphosaurus* (Fig. 3). These tooth apices also appear to bear enamel striations (Fig. 3).

The right jugal, present on CM 93778, is elongate and mediolaterally narrow, with the Y-shaped posterior end forming the anteroventral margin of the temporal fenestra and ventral margin of the orbit (Fig. 1). The dorsal process of the jugal, which forms part of the postorbital bar, appears forked but this may be the result of crushing. The anterior portion of the jugal is thin and tapers to a point that would have contacted the dorsal edge of the posterior process of the maxilla. Overall, the jugal is very similar to that of other edaphosaurids including *Glaucosaurus, Gordodon*, and species of *Edaphosaurus*^22,23^.

CM 93779 preserves the left parietal (Fig. 2). The parietal is large, plate-like, and rectangular with emarginations and processes for the contacts with adjacent elements. Anteromedially, there appears to be an area that was overlapped by the frontal. Anterolaterally, the parietal bears a large embayment for contact with the postfrontal. Medially, there is a large circular excavation near the centre of the element, which formed part of the margin of a large pineal foramen. Posterolaterally, the parietal is embayed for contact with the supratemporal. Posteriorly, a gentle emargination receives both the tabular and part of the postparietal. Overall, the parietal morphology is not as derived as that of *Edaphosaurus* where the lateral portion of the parietal is ‘free’. Instead, the parietal morphology is similar to that of early-diverging sphenacomorphs and most similar to that of *Ianthasaurus* among edaphosaurids. There is also a bone that appears to be the right size and shape for a frontal, but is mostly obscured by the overlapping parietal, preventing definite identification.

Both the right pterygoid of CM 93778 and left pterygoid of CM 93779 are exposed in primarily ventral view (Figs. 1, 2). The pterygoid has a long anterior ramus similar to those in most species of *Edaphosaurus*^22,24,25^*.* Its anterior ramus bears at least three distinct fields of palatal teeth, which appear to be emplaced atop shallow bosses. The anteriormost teeth are slightly enlarged as in *Ianthasaurus*^26^. The microCT scans reveal that the palatal teeth are slightly bulbous and have pointed apices. The dorsoventrally shallow transverse flange on the pterygoid bears a distinct, dense patch of teeth. The structure of this flange appears more derived than the condition seen in *Ianthasaurus* and more similar to that of *Edaphosaurus*, perhaps representing an intermediate condition. The quadrate ramus of the pterygoid is flattened, tall, and fan-shaped posteriorly, similar to that of *Ianthasaurus*^26^.

The only braincase element confidently identified for *Melanedaphodon* is the parabasisphenoid exposed in dorsal view on CM 93779 (Fig. 2). This compound bone is robust and has a long cultriform process. The cultriform process bears a flattened extent of bone ventrally, which may be part of a crista ventrolateralis. There are two moderately developed basipterygoid processes, which undoubtedly would be more prominent ventrally. The posterior region of the parabasisphenoid is roughly triangular in outline and expanded posteriorly. Overall, the structure of the parabasisphenoid appears similar to that in other basal sphenacomorphs.

A nearly complete right mandibular ramus is preserved in CM 93778 in lateral view, missing only the anterior ends of the dentary and splenial (Fig. 1). The dentary appears shallow anteriorly but increases in depth posteriorly. A low coronoid process extends posteriorly from the posterodorsal end of the dentary. On the lateral surface of the dentary, close to the tooth row, there is an evenly spaced series of moderately-sized foramina that each open into a posteriorly directed sulcus. These openings likely transmitted both blood vessels and branches of the inferior alveolar nerve. The dentary preserves a partial tooth row comprising approximately 14 tooth spaces with 12 well-preserved teeth in place. The teeth are identical in morphology to those of the upper jaw but appear to be slightly smaller in size. A thin, elongated partial splenial embraces the ventral surface of the dentary. Posterior to the dentary, the lower jaw gently bows outward in a manner similar to that in *Gordodon*^27^. The surangular and angular meet the dentary along slightly interdigitated sutures. The surangular is long and gently tapers posteriorly. The lateral surface of the angular is larger than that of the surangular. The angular is massive, long, and quadrangular. Anteriorly, it has a process that extends along the dentary ventrally and appears to have reached the splenial. The articular has only a slight, irregularly shaped exposure in lateral view. The suture between the articular and angular is somewhat interdigitated.

CM 93779 preserves two vertebrae and a limb bone (Fig. 2). One vertebra appears to be a small, cylindrical distal caudal and the other is a dorsal lacking most of its neural spine. The dorsal vertebra is very similar to those of other edaphosaurids including *Edaphosaurus*, *Gordodon,* and *Ianthasaurus*^27,28^. The centrum appears hourglass-shaped with a moderately developed, rounded ventral keel. The neural arch is robust with prominent pre- and postzygapophyses exposed in ventrolateral aspect. The preserved portion of the neural spine, separated from the centrum, slightly tapers distally. The limb bone has a small rod-like shaft and moderately developed diaphyses; it possibly represents a radius. It bears general resemblance to the radii of early ‘pelycosaurs’ (e.g., ophiacodontids^28^).

## Phylogenetic relationships

We explored the phylogenetic relationships of *Melanedaphodon hovaneci* using two distinct character–taxon matrices. In order to determine the large-scale phylogenetic relationship of *Melanedaphodon hovaneci* within amniotes we used the recent matrix of Ford and Benson^29^ (see Supplementary Information, S1, for matrix). Following this, we used a modified version of the character-taxon matrix by Spindler et al.^20^ (see Supplementary Information, S2, for matrix), which is the most up-to-date data matrix for assessing the interrelationships of Edaphosauridae. For this analysis, in addition to our new taxon, we also added the recently described *Gordodon*^27^ as well as limited our OTU sample to taxa that were reasonably well-diagnosed and not solely based on highly fragmentary postcranial material. In both analyses *Melanedaphodon* was coded as a composite of both specimens to achieve the most complete character sampling.

**Figure 4:**
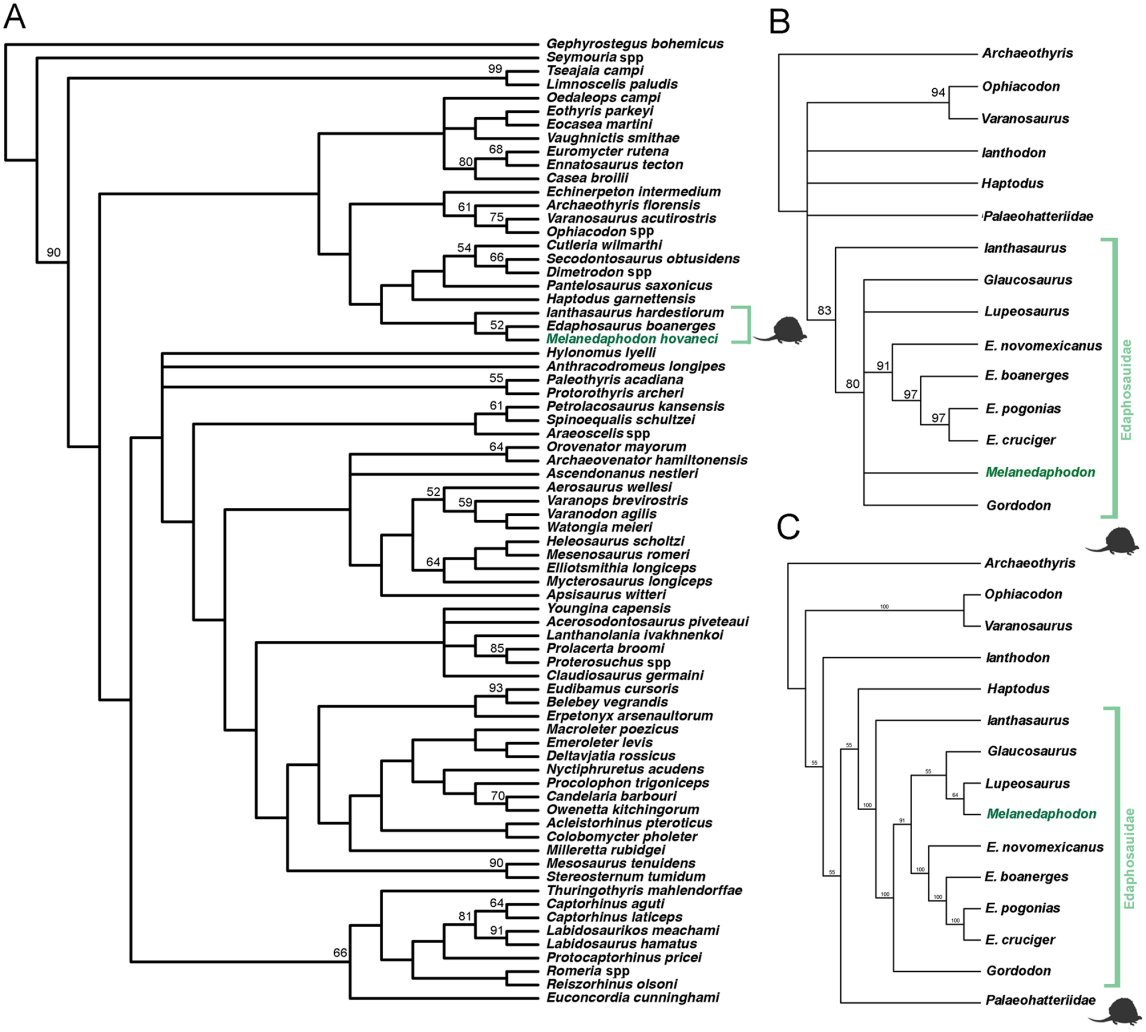
(**A**) Strict consensus of the results of the parsimony analysis of early amniotes based on Ford and Benson^29^. (**B**) Strict consensus of the results of the parsimony analysis of Edaphosauridae based on Spindler et al.^20^. (**C**) Majority-rule consensus of the results of the parsimony analysis of Edaphosauridae based on Spindler et al.^20^. In all analyses, Edaphosauridae is demarcated by a light green bracket and *Melanedaphedon hovaneci* gen. et sp. nov. is highlighted in dark green. In the strict consensus trees, bootstrap values greater than 50% are indicated above nodes.

For the first analysis, using the matrix of Ford and Benson^29^, we conducted a parsimony analysis using PAUP software v4.0b10^30^ with *Gephyrostegus* designated as the outgroup. The heuristic search option was selected, with Maxtrees set at 10,000 and set to automatically increased by 100. All characters were treated as equally weighted, and multistate taxa were treated as polymorphic. Ambiguous character states were resolved using the ACCTRAN setting. Indices of goodness of fit of the character data to the topology (e.g., consistency index [CI], homoplasy index [HI]), retention index [RI], rescaled consistency index [RC]) were calculated in PAUP. To assess support of internal nodes, bootstrap values were calculated using the “fast” stepwise addition option. The parsimony analysis recovered 18 most parsimonious trees (MPT), each with 1619 steps (CI = 0.255; HI = 0.772; RI = 0.593; RC = 0.151). The strict consensus of the results recovered *Melanedaphodon* as an edaphosaurid synapsid, specifically as sister taxon to *Edaphosaurus* to the exclusion of *Ianthosaurus* (Fig. 4A). The topology otherwise remain consistent to what is reported in Ford and Benson^29^.

Next, using the matrix of Spindler et al.^20^, we performed a parsimony analysis using PAUP software v4.0b10^30^ with the early ophiacodontid *Archaeothyris* specified as the outgroup. We used the branch-and-bound search option. Maxtrees were set at 10,000 and automatically increased by 100, all characters were equally weighted, and all multistate taxa were treated as polymorphic. All ambiguous character states were resolved using the ACCTRAN setting. Indices of goodness of fit of the character data to the topology were calculated in PAUP. To assess support of internal nodes, bootstrap values were calculated using the full heuristic search option with 100 replicates. The parsimony analysis recovered 11 most parsimonious trees (MPT), each with 120 steps (CI = 0.683; HI = 0.317; RI = 0.819; RC = 0.560). The strict consensus of the results recovered *Melanedaphodon* within a polytomy of derived edaphosaurids comprising *Glaucosaurus*, *Gordodon*, *Lupeosaurus*, and a clade of *Edaphosaurus boanerges*, *E*. *cruciger, E*. *novomexicanus,* and *E*. *pogonias* (Fig. 4B). This polytomy is recovered as the sister clade to the early edaphosaurid *Ianthasaurus*. Our analysis found *Ianthosaurus* as the basalmost member of Edaphosauridae. The Majority–rule consensus of the results recovered a clade consisting of *Melanedaphodon* as sister-taxon to *Lupeosaurus* to the exclusion of *Glaucosaurus* (Fig. 4C). This clade is recovered a sister-taxon to *Edaphosaurus* and its species to the exclusion of *Gordodon*. Again, *Ianthosaurus* is recovered as the earliest diverging edaphosaurid. Bootstrap values supporting these relationships are shown above nodes in Fig. 4.

## Discussion

**Figure 5:**
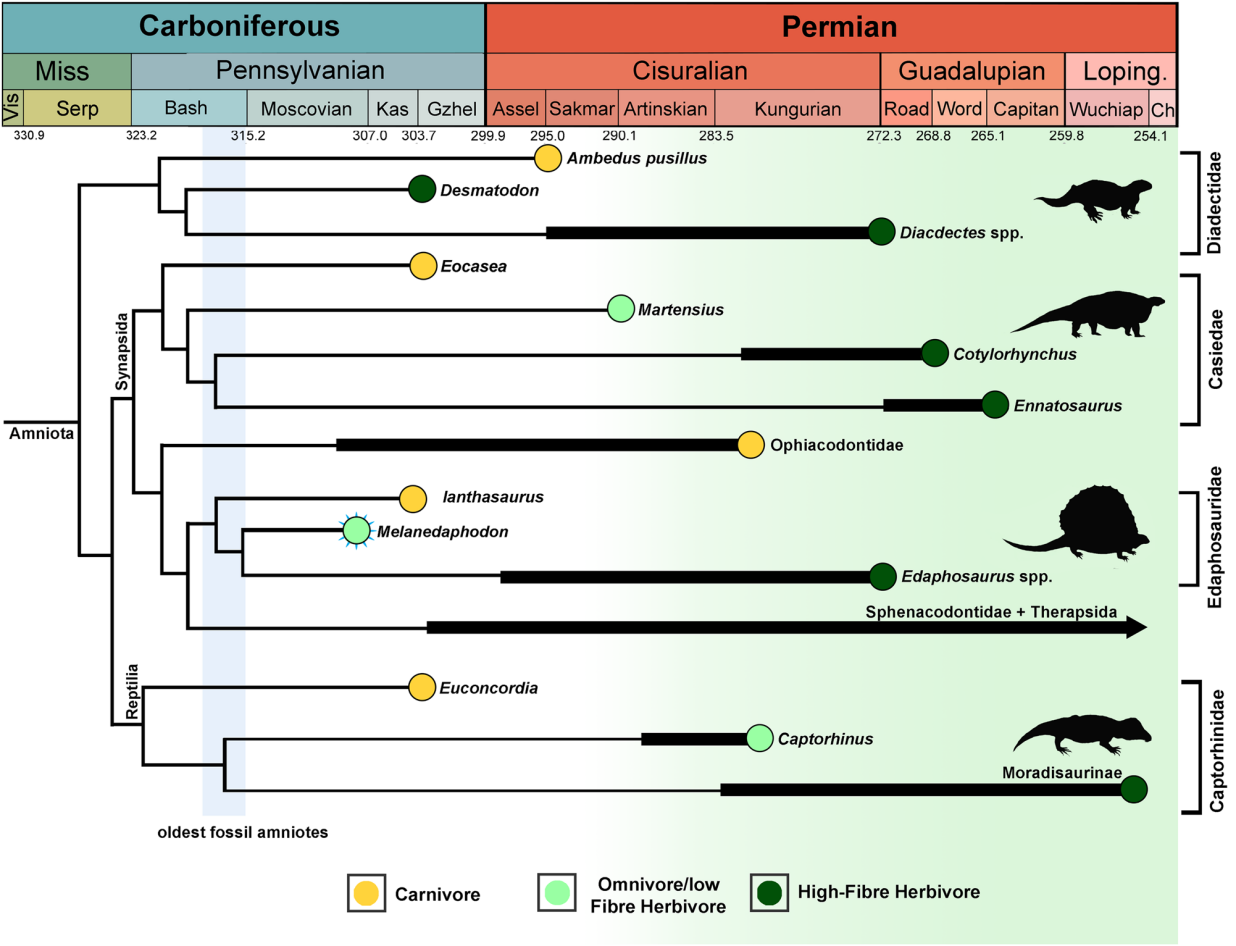
Time-calibrated phylogeny showing the origins of major clades with herbivory across the Permo-Carboniferous. Herbivorous/omnivorous feeding habits among tetrapods are shown to originate in the Late Carboniferous with *Melanedaphodon* as the oldest known example. However, herbivory becomes more widespread among tetrapods in the Early Permian with a greater volume of high-fibre herbivores. *Melanedaphodon* is indicated with turquoise sunburst pattern. Dietary inferences are colour-coded (see figure legend).

It has repeatedly been hypothesized that herbivory arose independently in several tetrapod clades around the Permo-Carboniferous boundary and became more widespread during the Permian^2,3,5^. The earliest tetrapod groups adopting herbivory—diadectids, edaphosaurids, and captorhinids—have their origins in the Late Carboniferous but did not diversify until the early Permian (Fig. 5). High-fibre herbivory is thought to have evolved in these clades based on suites of morphological characters correlated with herbivory in extant reptiles and mammals. These herbivorous adaptations include the presence of occluding teeth or tooth batteries (marginal and/or palatal), tooth-wear patterns, modifications of the jaw apparatus for oral processing of plant material, and expansion of the thorax and abdomen (as documented by the dimensions of the ribcage) to accommodate large guts housing microbial endosymbionts to facilitate digestion of cellulose^2,3,5^.

**Figure 6:**
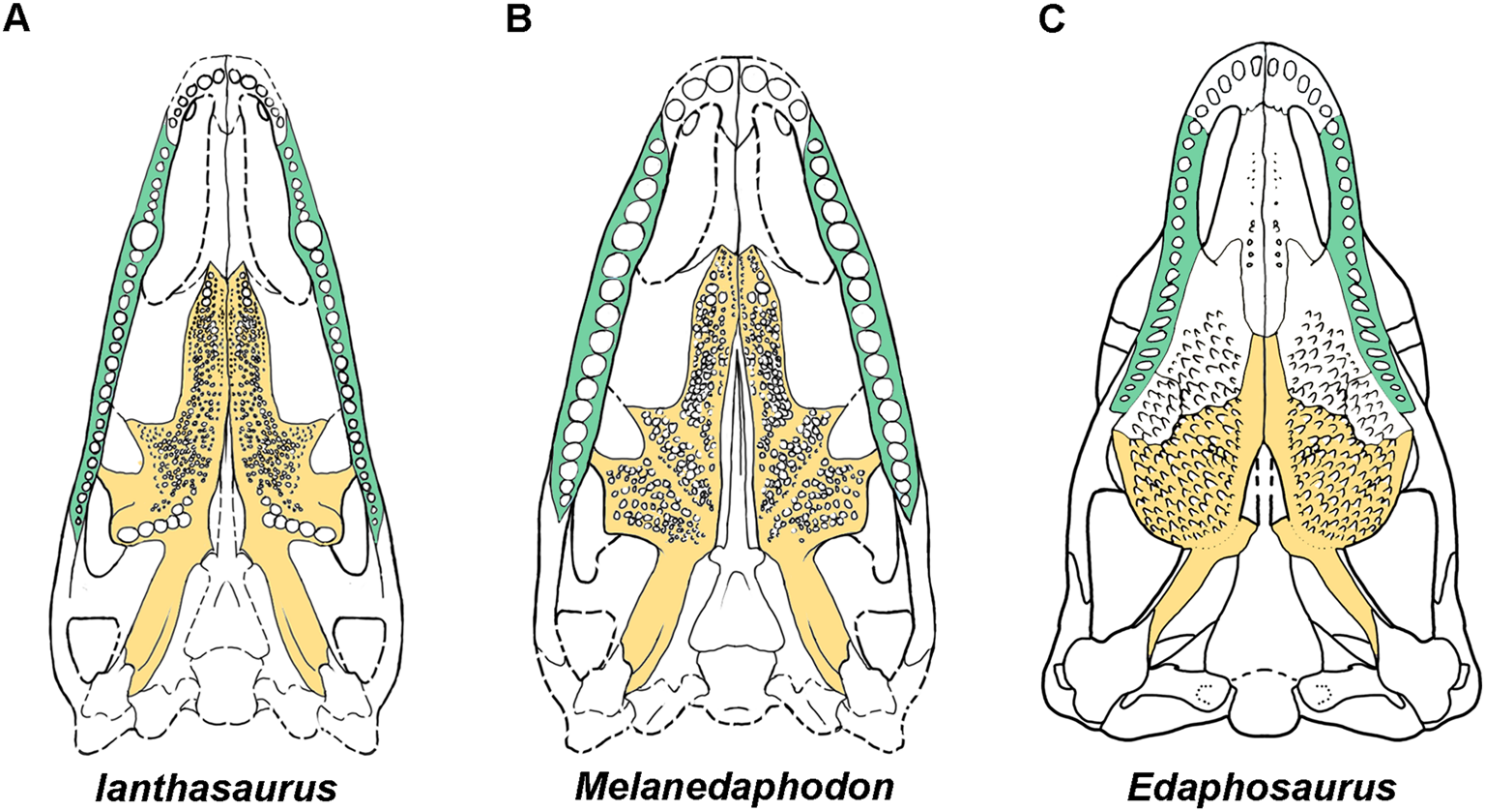
Comparative palatal reconstructions of three edaphosaurids. (**A**) Palate of the Late Carboniferous (Kasimovian) early edaphosaurid, *Ianthasaurus hardestiorum*, from Garnett, Kansas (based on observations of ROM 59933). (**B**) Palate of the Late Carboniferous (Moscovian) edaphosaurid, *Melanedaphodon hovaneci* gen. et sp. nov., from Linton, Ohio. (**C**) Palate of the Early Permian edaphosaurid, *Edaphosaurus boanerges*, from Archer County, Texas^25^. Maxilla and pterygoid are highlighted in green and yellow, respectively.

The Moscovian-age *Melanedaphodon* is the oldest known edaphosaurid synapsid, and unlike the late Kasimovian-age edaphosaurid *Ianthasaurus,* which was likely a carnivore, it already presents a craniodental structure more similar to that of *Edaphosaurus*^25^(Fig. 5). The pterygoid of *Melanedaphodon* shows a poorly developed transverse flange that lacks the single large tooth row found in *Ianthasaurus*. Instead, the entire pterygoid (and likely the whole palate) is covered by a moderately-developed tooth battery, which appears intermediary towards the condition seen in *Edaphosaurus* (Fig. 6). Due to preservation, there is as yet no information regarding wear on the palatal teeth of *Melanedaphodon,* but it is likely that the palatal dentition could have served in processing plant material, though perhaps not as extensively as in the high-fibre herbivore *Edaphosaurus*^28^ (Fig. 6). The large and bulbous marginal teeth are is reminiscent of a tooth morphotype more often associated with feeding on hard-shelled invertebrate material such as arthropods or molluscs (durophagy) or on seeds (granivory)^31,32^. Furthermore, similar types of bulbous teeth can be found in present-day squamates (e.g., *Tiliqua rugosa*, *Tiliqua multifasciata*, *Dicrodon guttulatum*, *Tupinambis rufescens*) that also consume plant material and are varying degrees of omnivorous or herbivorous^31–36^. In *Melanedaphodon*, the combination of the structure of the marginal teeth along with the palatal dentition suggests that plant material made up a considerable portion of the diet. However, for *Melanedaphodon*, we cannot eliminate the possibility that it fed on invertebrates in addition to plant material; therefore, it is best considered an omnivore–low-fibre herbivore that was capable of exploiting plant resources.

*Melanedaphodon* provides the oldest known record of probable omnivory–low-fibre herbivory in amniote evolution, and thus provides novel data for our understanding when herbivory originated (Fig. 5). *Melanedaphodon* also reveals that herbivory in synapsids significantly predated the origin of this feeding strategy in the conventional configuration of traditionally recognized reptiles (i.e., without the addition of recumbirostrans sensu Pardo et al.^37^) (Fig. 5). Among reptiles, early-diverging captorhinid eureptiles evolved different strategies to expand their feeding habits, such as the early Permian *Captorhinus*^5^ that had single or at most a few longitudinal rows of teeth in the maxilla and dentary and have long been considered omnivorous. Larger, more derived, captorhinids show craniodental features that suggest high-fibre herbivory including broad maxillae and dentaries with up to 11 longitudinal rows of isodont teeth, which show tooth wear^38^. The bulbous marginal teeth of *Melanedaphodon* provide a previously unrecognised dental morphotype among the earliest synapsids^39,40^, being clearly adapted for feeding on tough, resistant food stuffs. Whereas such teeth are rare among early synapsids, similar types of bulbous teeth are present in other Permo-Carboniferous groups of tetrapods, including the possible captorhinid reptile *Opisthodontosaurus,* but more frequently among recumbirostran ‘microsaurs’. Recumbirostrans, which have recently been reinterpreted as a fossorially adapted group of early reptiles^9,37,41–44^, apparently present the greatest dental variety among early tetrapods, comparable to that of present-day squamates^45^. Dentitions adapted for durophagy in recumbirostrans appear in the fossil record as early as the Bashkirian (~ 318 Ma)^46,47^. It is possible that durophagous feeding habits provided an alternate pathway to herbivory with bulbous teeth providing an exaptation to facilitate oral processing of the great variety of tougher plant food available in the Late Pennsylvanian. Particularly, some groups of ‘microsaurs’ such as pantylids have apparently developed similar palatal structures to those of edaphosaurids, but with durophagous tooth morphology on the palatal and marginal dentition^48,49^. Such teeth would have been suited for processing roots and tubers, seeds or megaspores, but additional research into the systematics and anatomy of ‘microsaurs’ is needed to confirm this hypothesis.

## Conclusions

**Figure 7:**
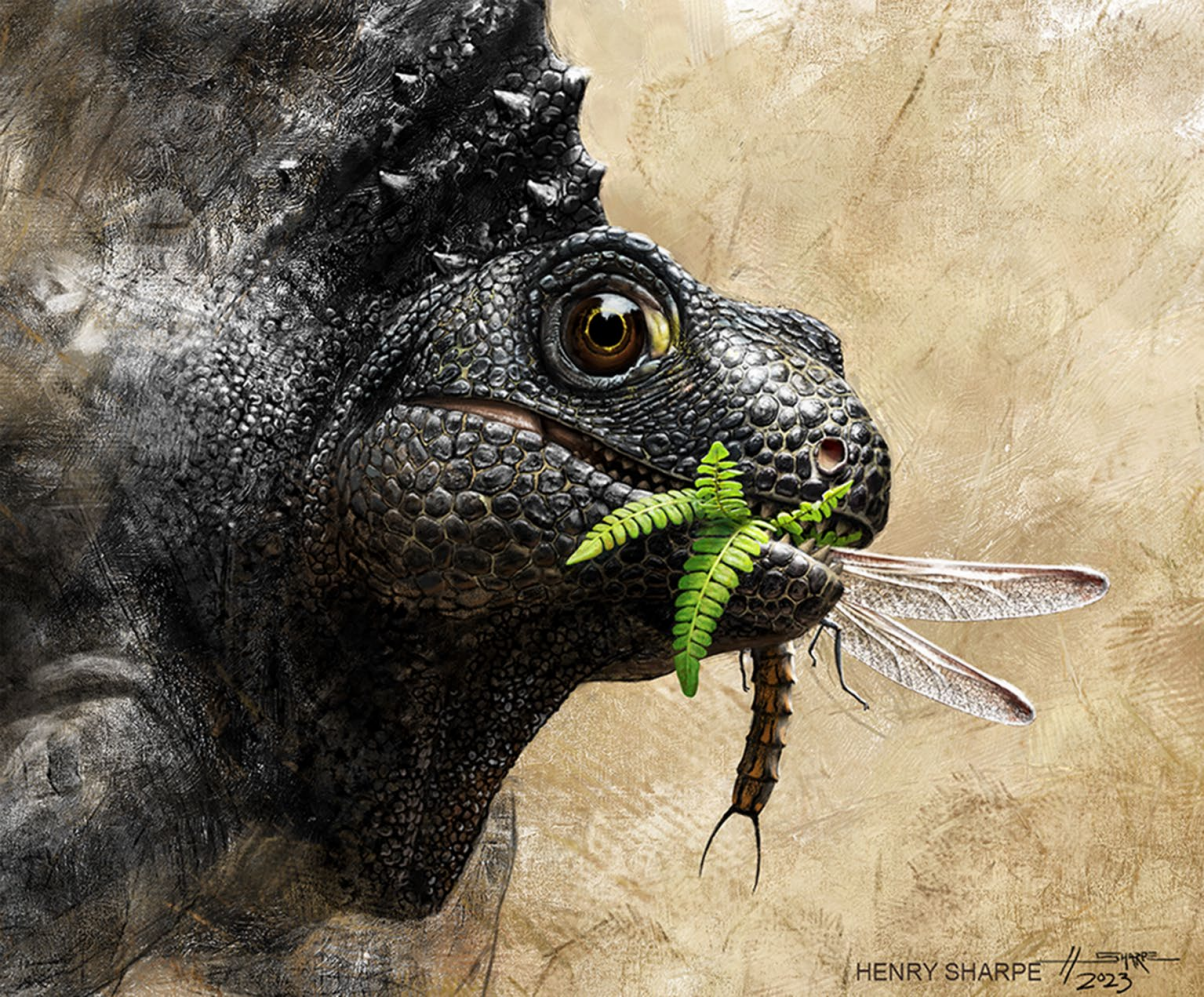
Life reconstruction of *Melanedaphedon hovaneci* gen. et sp. nov. (created by Henry Sutherland Sharpe).

*Melanedaphodon hovaneci* gen. et sp. nov., from the Middle Pennsylvanian, Linton, Ohio, provides the oldest known record of probable omnivory–low-fibre herbivory in amniote evolution, and offers new anatomical data in understanding when and how herbivorous adaptations arose (Fig. 7). *Melanedaphodon* is the oldest known occurrence of an edaphosaurid, firmly establishing the presence of this clade in the Moscovian (~ 307 Ma; Fig. 5). Finally, the bulbous marginal dentition of *Melanedaphodon* represents a previously unrecognised dental morphotype among the earliest synapsids, indicating some form of durophagous omnivory may have provided an intermediate condition between carnivory and high-fibre herbivory.

## Supplementary Information

Supplementary Information.

## Data Availability

All phylogenetic data used in this study, including the matrices, are provided in the accompanying Supplementary Materials. CT scans of CM 93778 and CM 93779, only provide surface details due to the nature of preservation in cannel coal. Stereolithographic files of the scans can be provided upon request to the corresponding author. This published work and the nomenclatural acts it contains have been registered in ZooBank, the proposed online registration system for the International Code of Zoological Nomenclature (ICZN). The ZooBank LSIDs (Life Science Identifiers) can be resolved and the associated information viewed through any standard web browser by appending the LSID to the prefix http://zoobank.org/. The LSIDs for this publication are: urn:lsid:zoobank.org:pub:5EF205BE-1C54-4AA6-9F13-C0F8D0EEC161 (article); urn:lsid:zoobank.org:act:85C2C125-C717-4519-B82F-9226D9D9F51B (genus); and urn:lsid:zoobank.org:act:E57B0D8C-B076-4B73-AF37-602C33DCC09C (species).

## References

[1] Olson EC (1966). Community evolution and the origin of mammals. Ecology.

[2] Sues H-D, Reisz RR (1998). Origins and early evolution of herbivory in tetrapods. Trends Ecol. Evol..

[3] Reisz RR, Sues H-D, Sues H-D (2000). Herbivory in late Paleozoic and Triassic terrestrial vertebrates. Evolution of herbivory in terrestrial vertebrates: perspectives from the fossil record.

[4] Beerbower R, Olson EC, Hotton N (1992). The early development of tetrapod herbivory. Paleontol. Soc. Spec. Publ..

[5] Hotton NIII, Olson EC, Beerbower R, Sumida SS, Martin KLM (1997). Amniote origins and the discovery of herbivory. Amniote origins.

[6] Reisz RR, Berman DS (1986). *Ianthosaurus hardestii* n. sp., a primitive edaphosaur (Reptilia, Pelycosauria) from the Upper Pennsylvanian Rock Lake Shale near Garnett, Kansas. Can. J. Earth Sci..

[7] Modesto SP, Reisz RR (1990). A new skeleton of *Ianthasaurus hardestii*, a primitive edaphosaur (Synapsida: Pelycosauria) from the Upper Pennsylvanian of Kansas. Can. J. Earth Sci..

[8] Reisz RR, Fröbisch J (2014). The oldest caseid synapsid from the Late Pennsylvanian of Kansas, and the evolution of herbivory in terrestrial vertebrates. PLoS ONE.

[9] Mann A, McDaniel EJ, McColville ER, Maddin HC (2019). *Carbonodraco lundi* gen. et sp. nov., the oldest parareptile, from Linton, Ohio, and new insights into the early radiation of reptiles. R. Soc. Open Sci..

[10] Mann A, Dudgeon TW, Henrici AC, Berman DS, Pierce SE (2021). Digit and ungual morphology suggest adaptations for scansoriality in the late Carboniferous eureptile *Anthracodromeus longipes*. Front. Earth Sci..

[11] Brocklehurst N, Kammerer CF, Benson RJ (2020). The origin of tetrapod herbivory: Effects on local plant diversity. Proc. R. Soc. B.

[12] Brocklehurst N, Benson RJ (2021). Multiple paths to morphological diversification during the origin of amniotes. Nat. Ecol. Evol..

[13] Hook RW, Ferm JC (1985). A depositional model for the Linton tetrapod assemblage (Westphalian D, Upper Carboniferous) and its palaeoenvironmental significance. Philos. Tran. R. Soc. Lond. B Biol. Sci..

[14] Hook RW, Baird D (1988). An overview of the Upper Carboniferous fossil deposit at Linton, Ohio. Ohio J. Sci..

[15] Carroll RL, Baird D (1972). Carboniferous stem-reptiles of the family romeriidae. Bull. Mus. Comp. Zoolog..

[16] Osborn HF (1903). On the primary division of the Reptilia into two sub-classes, *Synapsida* and *Diapsida*. Science.

[17] Ivakhnenko MF (2003). Eotherapsids from the East European Placket (Late Permian). Paleontol. J..

[18] Spindler F, Scott D, Reisz RR (2015). New information on the cranial and postcranial anatomy of the early synapsid *Ianthodon schultzei* (Sphenacomorpha: Sphenacodontia), and its evolutionary significance. Fossil Record.

[19] Cope ED (1882). Third contribution to the history of the Vertebrata of the Permian formation of Texas. Proc. Am. Philos. Soc..

[20] Spindler F, Voigt S, Fischer J (2020). Edaphosauridae (Synapsida, Eupelycosauria) from Europe and their relationship to North American representatives. Paläontol. Z..

[21] Falconnet J (2012). First evidence of a bolosaurid parareptile in France (latest Carboniferous-earliest Permian of the Autun basin) and the spatiotemporal distribution of the Bolosauridae. Bull. Soc. Géol. France.

[22] Case EC (1906). On the skull of *Edaphosaurus pogonias* Cope. Bull. Am. Mus. Nat. Hist..

[23] Modesto SP (1994). The Lower Permian synapsid *Glaucosaurus* from Texas. Palaeontology.

[24] Modesto SP, Reisz RR (1992). Restudy of Permo-Carboniferous synapsid *Edaphosaurus novomexicanus* Williston and Case, the oldest known herbivorous amniote. Can. J. Earth Sci..

[25] Modesto SP (1995). The skull of the herbivorous synapsid *Edaphosaurus boanerges* from the Lower Permian of Texas. Palaeontology.

[26] Mazierski DM, Reisz RR (2010). Description of a new specimen of *Ianthasaurus hardestiorum* (Eupelycosauria: Edaphosauridae) and a re-evaluation of edaphosaurid phylogeny. Can. J. Earth Sci..

[27] Lucas SG, Rinehart LF, Celeskey MD (2018). The oldest specialized tetrapod herbivore: A new eupelycosaur from the Permian of New Mexico, USA. Palaeontol. Electron..

[28] Romer AS, Price LI (1940). Review of the Pelycosauria. Geol. Soc. Am. Spec. Pap..

[29] Ford DP, Benson RB (2020). The phylogeny of early amniotes and the affinities of Parareptilia and Varanopidae. Nat. Ecol. Evol..

[30] Swofford, D. L. (2002). PAUP: phylogenetic analysis using parsimony (and other methods), version 4.0 beta. http://paup.csit.fsu.edu/.

[31] Estes R, Williams EE (1984). Ontogenetic variation in the molariform teeth of lizards. J. Vertebr. Paleontol..

[32] Shea G (2006). Diet of two species of bluetongue skink, *Tiliqua multifasciata* and *Tiliqua occipitalis* (Squamata: Scincidae). Aust. Zool..

[33] Hutchinson MN (1993). Family Scincidae. Fauna Aust..

[34] Van Leeuwen JP, Catenazzi A, Holmgren M (2011). Spatial, ontogenetic, and sexual effects on the diet of a teiid lizard in arid South America. J. Herpetol..

[35] Juri GL, Naretto S, Mateos AC, Chiaraviglio M, Cardozo G (2015). Influence of life history traits on trophic niche segregation between two similar sympatric *Tupinambis* lizards. S. Am. J. Herpetol..

[36] Norval G, Gardner MG (2020). The natural history of the sleepy lizard, *Tiliqua rugosa* (Gray, 1825)—Insight from chance observations and long-term research on a common Australian skink species. Austral Ecol..

[37] Pardo JD, Szostakiwskyj M, Ahlberg PE, Anderson JS (2017). Hidden morphological diversity among early tetrapods. Nature.

[38] Dodick JT, Modesto SP (1995). The cranial anatomy of the captorhinid reptile *Labidosaurikos meachami* from the Lower Permian of Oklahoma. Palaeontology.

[39] Mann A, Paterson RS (2020). Cranial osteology and systematics of the enigmatic early ‘sail-backed’ synapsid *Echinerpeton intermedium* Reisz, 1972, and a review of the earliest ‘pelycosaurs’. J. Syst. Paleontol..

[40] Mann A, Reisz RR (2020). Antiquity of “sail-backed” neural spine hyper-elongation in mammal forerunners. Front. Earth Sci..

[41] Mann A, Maddin HC (2019). *Diabloroter bolti*, a short-bodied recumbirostran ‘microsaur’ from the Francis Creek Shale, Mazon Creek, Illinois. Zool. J. Linn. Soc..

[42] Mann A, Calthorpe AS, Maddin HC (2021). *Joermungandr bolti*, an exceptionally preserved ‘microsaur’ from the Mazon Creek Lagerstätte reveals patterns of integumentary evolution in Recumbirostra. R. Soc. Open Sci..

[43] Mann A, Pardo JD, Maddin HC (2022). Snake-like limb loss in a Carboniferous amniote. Nat. Ecol. Evol..

[44] Mann, A., Pardo, J. D., & Sues, H. D. 2022. Osteology and phylogenetic position of the diminutive ‘microsaur’ Odonterpeton triangulare from the Pennsylvanian of Linton, Ohio, and major features of recumbirostran phylogeny. *Zool j. linn. soc.* (early view).

[45] Melstrom KM (2017). The relationship between diet and tooth complexity in living dentigerous saurians. J. Morphol..

[46] Pardo JD, Mann A (2018). A basal aïstopod from the earliest Pennsylvanian of Canada, and the antiquity of the first limbless tetrapod lineage. R. Soc. Open Sci..

[47] Mann A, Gee BM, Pardo JD, Marjanović D, Adams GR, Calthorpe AS, Maddin HC, Anderson JS (2020). Reassessment of historic ‘microsaurs’ from Joggins, Nova Scotia, reveals hidden diversity in the earliest amniote ecosystem. Pap. Palaeontol..

[48] Romer AS (1969). The cranial anatomy of the Permian amphibian *Pantylus*. Breviora.

[49] Carroll RL, Gaskill P (1978). The order Microsauria. Mem. Am. Philos. Soc..

