# Supplementary material for: A new Carboniferous edaphosaurid and the origin of herbivory in mammal forerunners
Source: Sci Rep. 2023 Apr 5;13:4459. doi: 10.1038/s41598-023-30626-8 (PMC10076360; doi:10.1038/s41598-023-30626-8)
Supplement: Supplementary file 1 — Supplementary Information. [file 41598_2023_30626_MOESM1_ESM.docx]

**Supplemental Information**

**A new Carboniferous edaphosaurid and the origin of herbivory in mammal forerunners**

Arjan Mann^1*^, Amy C. Henrici^2^, Hans-Dieter Sues^1^, *and* Stephanie E. Pierce^3^.

*Format for* *Scientific Reports*

^1^Department of Paleobiology, National Museum of Natural History, Smithsonian Institution, MRC 121, P.O. Box 37012, Washington, D.C. 20013-7012, United States; Corresponding author* email: MannArjan@si.edu

^2^Section of Vertebrate Paleontology, Carnegie Museum of Natural History

4400 Forbes Avenue, Pittsburgh, PA 15213, United States;

^3^Museum of Comparative Zoology and Department of Organismic and Evolutionary Biology, Harvard University, Cambridge, MA 02138, United States.

**S1.** Modified taxon-character matrix of Ford and Benson (2020) that was used in this study to explore the large-scale phylogenetic relationships of *Melanedaphodon hoveneci*.

MATRIX

Gephyrostegus_bohemicus

00030000000000-000000?00000-030----0??0000200-000000?0100??000--1010000?0-0000-0-000000000-?0?000-0000000??0000??????????0000??00??00?0-000000000?1?00000-?0?1?????010010????001110000??00000?0??000-?01?0000??0000000000-00?????00??????0???-????????????0?0????????????????????????0?????-??-0-??????

Seymouria_spp

00000000?00000-000000001200-01110000??0001-00-010001?000010000--0000000?0-0000-(0 2)-000001000-00?00(1 2)-0000000?00000?1000????000000000000011000100001000?000?10000??1?1-01001010000011110000?0000030????10?01?0??00?0(0 1)010?01?0-0010100000000012010-000000000?000000000?1100000000010?0?00?0?011?-00-0-00100?

Tseajaia_campi

001-10000001?10001?10?00000-01111000???011-10-010000001000??00--111000001-0000-0-000001100-1??012-0001020?00(0 1)01?001-000002000??000101?0-111000000?0?00100-10010000?0010000---0011001??0?10110000000??0010000??100000010???0010101000001?00?1200000000000000000100111000000010??020000000??0-00-0-???0??

Limnoscelis_paludis

001-1001?00110-001011200000-01100000???011-10-010000001000??00--111011001-0000-1-000001100-000001-000100000010000000??0002?00??00011101101000101?10?00100?000000?0?0010000---001101000011011000??000-0010000???00?0?00000-0010101000?02?011020000000000?000000000111?1000001011010000000010-?0-0-??001?

Oedaleops_campi

100?21-0000111000??11?00100-011111000-1101-00-001001000100-000010110111010010?-01000001011000?011-00?10??0?01000???????????????????????????????????????????0???0????0?????????????0?????????????????????????????????0??1??0???0?????????0000201-100???0?10000??002????????000??100?1?00001?????????????

Eothyris_parkeyi

10??21-00001110001011?00100-01111101??1100100-00100101-100-000010110110010010110000000101100?0011-000100000000001300??1?001????000??1??????0010??10?????0-000100???0001000---0????00?1??1?1??0000000-??????????????????????????????????????????????????????????????????????????????????????????????????

Vaughnictis_smithae

?01-?0000001???????11???????0????????????01????????1?1-10????0???1?011101001010001?00010111???011-00?10????????????????????01??????01???01?000????0?????????????????0??00????0????00???????10?0????0-???????????????100???????0??0?????????????????????????????????????????????????1?00001?????????????

Eocasea_martini ??012???0???????????????????0????????????0??????????????????????????11001??10???01?0??????????0??-00110??0000010?????????0??????????????????????????????????0100?0?????000---0???????????????00??00???000?20?0000??0000?0-000?0??000001????????????????????????????????????0011(0 1)0001?0??0????1?0???0???

Euromycter_rutena

100121-0001110-0010002002010010----00-1100100-01100000010??0000100?011101001011001000010100000010-00110?0?0010011300??1011111?00000?????01?0001?010??0??0-000100100100???0---0?????1??010???0?100?????0?0020??00001???????????????0?????000?20?????????????????01210000001?????????????????????????????

Casea_broilii

100121-0001110-000000?00100-010----00-1100100-011?000?-10??0000?0??01??010010110010000?010000?010-00110?0??010001300??10111010000??01?0-01200010010?0???0-00?1001000001000---0001?11??001?110010000??0010000?000001000110-00000001010110??00201-0?0100001000200?010100000100?201000000001110010110?000?

Ennatosaurus_tecton

100221-0001110-0000112002010000----00-1100100-001000?01100-?001000001111100101101100000010?0?0010-001101?0?00001131-??1011??1??00??0110-01?00110110010010-2001?0???1001000---000111100010?11001??000?0????20?00???1?001???00?0?0?101??????00201-0???0000??0020???1?1???001?????100???0?????0010100?000?

Echinerpeton_intermedium

?????1-?000???????????011???0111100?1000???0?????0????????????????????????010??????????????????????????????????????????????????????????????????????????????????????21?????????????0?1?????????????????????00??100??011?1??00?100?0????????0120??????????100????????????????????0???0???1?????11????????

Archaeothyris_florensis

0?0?200?100??0-???????011???0211100?10??0??0?-0?0????011??????1?1210????100100-1??000010111?0?012-0??1?????01?????????1???????????????????????????????????????0??0?21????????1????0?0???1?????????????0???0100100??011110-000?0?00???0?0??????????????0?100000?????????0???002???0??00?1???????1???????

Varanosaurus_acutirostris

01022000000020-110111301100-03111011110001-10-020011?01100-0111012100110100100-110010010111001012-01010000000000021-??0100200010011011??11200200100?10100-1001?00002100000---1001100001?111?111??0010??00000?01?????01110-001110??00?01????????????100?1??????1???????????0002101??0?0?1001001011????1?

Ophiacodon_spp

011-2000(0 1)00020-100011?02100-03100010110001-10-02000?001100-0111112101110100100-110010010111001012-01010000001000020-000100200011010?110-1120020?110110?00-1001000012100000---10011000010101111100001000000010010001003110-100101000000300001(1 2)100010100011002001102000100010000101000(0 1)00000000100100111?

Cutleria_wilmarthi

011-?0010101?????10??????0??0?111000??0?0??0???2?1?1??110??0??1?201001101?0100-?1100???0110?0?012-01?10?0?1010?????????????????????????????????????????????1?0?????01?011????01---10?????????2???000-?0??1010?0100?0?1?1??10?????00?????10????01??01?0??10?0?????0?0???????????????????????????????????

Pantelosaurus_saxonicus

011-2100000000-0?1001?0110??01101000??0001-10-0?001??0110??0??111000011?1?0100-?100010-0110?0?011-01?10????0???????????????????????01???01?0??????0???????0100?0???0100101???0????10?0???????2?????????1???????10??????10-100?2??00??1??1?0?21??0?01?00?10000??000??0000?1?01??101?0????????01?????????

Haptodus_garnettensis

01021000000000-001011301100-01111010100001-10-000011001100-0101111100010110100-1110010-0110000002-01010?0?1????0?00???00?20000100100110-012001001001101?0-010000001000011100201---1010000?11011??000-1?0?0010010000000110-100020000??11?10012101010110011000001001000?001100??0101?00001???0?11110?0?1?

Secodontosaurus_obtusidens

011-1001000000-001011302100-01111010100001-10-0201-1001100-0101120200010100110-1110010-01100001120010103011010000100??0002?00011010111??01200200100110100-000000001210010111201---0010100111121??00?-10?0111?1200??012?10-2000210?01??????????01000110?11?000??????????????????101?0?10101???1?1???????

Dimetrodon_spp

011-1001110100-001011302100-01111001100001-10-0201-1001100-0101120201110100110-1100010-0110000002-010103011010000100??00020000110101111101200200100110000-010000001010010111201---1000000011121??000-1?00112?(0 1)210010(0 1)2120-2000210001?130130(0 1)1-0100011001100000(1 2)1020001001100110101000(0 1)01010001111?0111?

Hylonomus_lyelli

001-01-0000000-00001??00100-031100000-0001-00-00000000010??00010000000001-0000-0-100000000-0??00?-???101???0000??????????0011???0?001???010000????00??????00????00?210????????????0?0?1????10????????00000010??0?000???00-000?0?0?0??0??10???????????00?110?3-????????????000?1000000??111???100001????

Anthracodromeus_longipes 0?022???????????????????????0?????????0???????????????????????????1?00001-0000-?-1??001000-00?001-0101010?000000021-??00????????????????????????????????0-???????????0????????????????????????????????000?0100?000100111??000?0?0000?0??????????????????11010?000????????1?????0??0?01??????11?0??1?00?

Paleothyris_acadiana

000221-0000000-000000?00100-031110100-0000100-000000000100-00?10101000001-0000-0-100001001-001002-01?1010100000???1-??10000(0 1)1??0000010??0100010?000000??0-00?10000?20000?0---0????0000?01?1213100000-0000001001000100100??00?0000001?0??101?20010?0?000?110000000210101??10??1102000011111101111101000?

Protorothyris_archeri

000221-0000(0 1)00-000?01?00100-02111010??0000000-00000000010???00101(1 2)1000001-0000-0-100001001-001001-01?1010?000000??1-??10000????00000111001000201000?000?0-0000?0?0?2000000---001010000101?1??00??000-00???0100?000101?0(0 1)??00??000001?0??12002101000?10????003-??????????????0??????????????????????????

Petrolacosaurus_kansensis

00022000000000-000011?001010031110000-0001-00-00000000010??0001011100010101100-0110000?0100101101001010101000000001-000000011??00000101101000111000000011000000000?2000000---0????011?10110210000000-00100000121011101010-00112?000?000102112101100110011100012102101001?101111010101112?1011111101011?

Araeoscelis_spp

001-21-0000000-1?0000200100-01111000??0001-00-000001000100-00010?20001101-1000-0??00001001-1001010010102010000000200??0002000000000?1??????0011?010?0000100100000010000010---01---001?100011101000?0-?00??100121010111010-00102?00000001??002101100111011100112?02?0100???0011002010111201011100101011?

Spinoequalis_schultzei

?????1-??00100-000011?????1?0?111?00?-0??1-0?????00????1???????0?10???????????????????1??001?????????????????????????????1??????00?????????0010???0?????????????0???00????????????01???0?11210?????????0????001?????00??????????100????20??021??1??????10?????20??????0??1?????0??1?1???????11?????????

Orovenator_mayorum

001-2000000010-1011002011011020----0110101-01000001201-101000110120?????111100-01?00???01?????102001?1???????????????????001100000001011011001011100000110?00?0?01-200000????00011001110101210000101100???????10?????????????????0?????????????????????????????????????????????????????????????????????

Archaeovenator_hamiltonensis

011-2000000100-0001002011011020----01?0101-01100000201-10??001101200111?110100-0110000101101??002-00?101?1?00?0?????????0010???0000?101?01100201?10000110-?0?000???20000010?00001100111010121000000??000??0000100000110110000?0?100100000010211-000?(0 1)??????????????????????1?1?020?0?112??1?1111101011?

Ascendonanus_nestleri

0?1-200000000??????00?0110??01110?000-0001-0??0(0 1)?0?200010???0?0??2?0010?110100-011000010110?1?002-01?101???0000???????????11????0?00????01?002????0??0????0001?????20?00?10??0????000????????0?????????0???0?0???????101??00??00100000???0?021??????0????1?11?0?00111?1111???00???0?01????1?11010??100?

Aerosaurus_wellesi

011-2011010110-000101?011010011000000-0?00200-0?000120010??00?10?2??????11010100111?0010100??0012-010?02?11?????000?000000111??0100?????????02???10????????0?200?0020?0??10000001100001?1????300010110000?010(0 1)1100100101??100?2?000001???2?02001?10100101000001101110?11010????0201000????00010110??11?

Apsisaurus_witteri ?????000000??????????????????????????????1???????????????????????????????1?100-?1110001?11?10????????1????????????????????1????00??????????0010??100?01?0?????0??????????????0???????????????00001011?0??????0100??0?101??0??12?0????00??0????1?0?0?00??1101011100???????????1?????111120??????????????

Heleosaurus_scholtzi

011-2011?10000-000?00?021111011100?0?-0001-0100101-2???10??00?101201110?110100-010100010110110012-01?1021?00000?00???????0111??000101???0110020??100?0??0-00?1?0???20?00?100?0????00????1?1??3100?????????0?001100001301??000???101??0???0?0??1-0?0?00???1?1112100111?01??001(1 2)?0?0???11????0?10?????1??

Mesenosaurus_romeri

011-2011110000-100100?02111001110000??0001-0100101-211-10100011012010101110100-010100010110110012-01?1021000000000????0000111??01010101101100110100000100-00010001-20000010000????0001101112130000011000??????1100?0?101??000???100??0??????????????00????0?????0111???1010?0?002?1??1????1?????????1??

Mycterosaurus_longiceps

011-2011?10100-?0010??02111?011000000?000000100101-21??101000110?2?1?11111010100101000101101??012-01?1????0010000?????0???11100000101???01?0010???0???????00?000000200000????0?????0??????1?1?1001011?0??????0110000010110100?2?1?????????????1-000??0????0?0???0?????????0012?020?0?1120??0?10?00?????

Elliotsmithia_longiceps

0?1-2011110??????????????1??0?11?00???0?000????????2??-1010?0110?2?11111110101101010001?110110012-010102?100?????0??????????????????1??????0020???0??0????00?100????00???100?0????0?1?????????0????????????00?????????????????????1????????????????????????????????????????????????????????????????????

Varanops_brevirostris

011-?011010010-0000013121010011100110-00002011010001000100-0011012001010110101001?100010000100002-01010200101?0???0000?100111?00000?1???????01???10?????0-10020000020102-100100011000?1?1011130?01011000??01?11100?0010111100?200000?0??02011-(0 1)1010100101000002?0111011?01001110001010020000010010??11?

Varanodon_agilis

111-(1 2)011010000-0?0?01?1210??01110011??00002011010001200100-001101211?0?0110101001100001000010?0?2-01?1???010????0000????????????????1???????02????0???????10?200???20102-10010????0?0????????3??010???00?101?1110010?101111001210000002?00??????0?0?????1000??0100?1??11010?????????????0???????????11?

Watongia_meieri

?????011010??0-??????????????????????????????????????????????????2?1??????????????????????????????????????????????????????????????????????????????????????????????????????????????????????????????????????????1?00?00101??100121????????00??????0?0????010000?1000110110?1?????????????????????????????

Youngina_capensis

001-1000000100-000000?02110-020-----??0001-00-0101-1?00100-1001012000110111100-0110000101001?110210101010000010?0001011000(0 1)000000?0011110100020?010000111000000001-200???????0????000?1?1?1??3?00??0-?01????00?000000?010-000100101?0012100?201-0100011?1101011110?11?11?1010100001111??00111100?01111?

Acerosodontosaurus_piveteaui

0????000000???????????0211??03????????00?1-??????1-?100100-??010?2???11??1?100-011?010??100??????????1??????????????????????????????1?????????????????????????00???????0010?1?????????????121????10???0100???01?0??00?01??(0 1)0010?100??0????????????????1???0011?110110000??1101?00??1?????0?????????????

Lanthanolania_ivakhnenkoi

00???00???0??0-???????0210100?0----0??000000?????1-2?0010??10010???0??101-?100-0110010??10???????????1????????????????????00100?????1????????????1???0??????????????0??0????????????0?????121??????????????????????????????????????????????????????????????????????????????????????????????????????????

Claudiosaurus_germaini

001-11-?000000-100000?01200-030----0??0001-00-1001-2?1-10??00011?2?0011?1-1100-0110010-010010?111101-11--0?1----001-??000?000??00000111101000011?10?001110?00000???2000000---0001100001010121?1????0-??11?0000?000101?010-000?0?1001000??00?21?????0001?11000101?011010(0 1)011??1?0??11011200101100101011?

Prolacerta_broomi

001-101101000110010002022011010----0110001-00-1201-2100100-1001012100010111100-0110010-01001101(0 2)1101-11--001----00011110001000000(1 2)00111101000201210000111000001011-20100011010????010010111213011??0-?111?00111001?10(0 1)110-00112110011002??11201-01000?2?1111012100??1?0??111010000101112?001110?111?11?

Proterosuchus_spp

001-1011110001110400020210110(1 2)0----0??0?00100-1201-2?0010??11?102(0 1 2)000110011100-11000(0 1)0-010011?1(0 2)1101-11--0?0100???0?11?0???0000??2001111011002012?00?0111010?11011-21100?11011????0100??1?121?011??0-?1?1???111101010011???0012?100110(0 2)2??10201-00000?2???1???2100???????110?1?0?0?1?11200???10??1101??

Thuringothyris_mahlendorffae

001-21-0000100-000001?00100-010----0??0?00100-000000?01100-00000000000101-0000-1-10000100?-001000-0101010100010???1-??00?00000??0??1111?01?0000?0?0?10?1??0??0?000???0???0---?????00?00????????????????10??00?10??10110???000?0?000???????0021????????1?10003-2001?10011?1?????????010?10???01?100110??

Captorhinus_aguti

100011-0000100-013011101100-1-1110000-0011-00-000000001101-00000000000001-0000-1-100000100-001000-01-11--1000100001-??000200(0 1)0000101111101200001000003--11000000001001100100000111012000111213100000-00100000010100010000-001010000000?1110020000001000010003-0?02110000?1010210200000001100010110100??

Captorhinus_laticeps

100011-0000000-012011101100-011110000-0011-00-000000001101-00000000000001-0000-1-100000100-001000-01-11--1000100001-??00020000?00101111101200001000003--1100000000?001100100000111012000111213100000-0011?????????0????00-?01?1?0000?0??????????????????????????????0???????????????????????0??????????

Labidosaurikos_meachami

000011-0000?00-012?11101100-1-0----0??0111-00-000000?0110??00001102000101-0000-1-100000100-001000-01-11--100?100001-?????2000??00101111111?00201?00??2--1110?00000100110010000011100?0011112131??010-0?????????????????????????????????????????????????????????????????????????????????????????????????

Labidosaurus_hamatus

000011-0010(0 1)00-013011201100-010----0??0011-00-000000?0110??00001001000001-0000-1-100000100-001000-01-11--1000100001-??0002000???0101111101200001000?02101110000000?0111001000001111020011112131??010-0??????????????????????1?1????0????????????????????????????????0???????????????????????0??????????

Protocaptorhinus_pricei

100001-0000000-012011101100-01111000??0010100-000000?0110??00000001000001-0000-0-100001001-001000-01-11--?000100001-???00???????????1??????0000???0?02--1100?00000?00010?10??0????0?0???1?12?3??????????0?????101?1????0???01?10?00????????021??????????1??03-??????0???????????????????????0??????????

Romeria_spp

100021-?000(0 1)00-012011100100-01111000??0010000-000000?0110??00001(0 1)(0 1)0000001-0000-0-000000000-001000-01-11--?000?00??1-???00200???????11?1001200001000?0?--1100?0?000?0001000---0????01100?1?121?1????0-???????????????????????1??????0????????????????????????????????0????1??????????????????0??????????

Euconcordia_cunninghami

?00021-1000000-010?00?00100-01100000???001-00-00000000110?-00000000000001-0000-1-100???00?-001000-01-11--10001000?1-?????001100000001???01000001010?00??11000??0001?00???0---0????000?1????2???????????????????????????????????????????????????????????????????????????????????????????????????????????

Reiszorhinus_olsoni

000?0000?10000-012011100100-01110000??1110000-00000000110?-00000101000001-0000-1-000000000-001000-01-11--1000000???-??????????????????????????????????????0000?0???0001000---0011010100?1112101000010??????????????????????????????????????????????????????????????????????????????????????????????????

Eudibamus_cursoris

??1-01-00001?????0?????????10?0----?????????????0??????1?11???10??????0??1?10??0???000??01??01????1--1??????????0??????????00????0?11???11?11-2???0??01???0????0???????310---?0????????????????????????1???????????????????0??10?-0000????????1??????0??10??01?????????0????????2?111111?000?111-?1110?

Mesosaurus_tenuidens

001-11-0000000-000-00001300-010----00-0?01000-0200-1300100-00000120000?01-0000-1-100001001-101011-0011010000001?001-??00?2100??000101?1101000000100001--??0000?0?0?20000?110001---00210?0?121?000000-??00???00(0 1)0?100100(0 1)0-100010100100010201201-1100002?1101110100?10?0000?000000001?11200100100101001?

Stereosternum_tumidum

001-1000000000-000?10002300-010----00-0?01000-0200-????100-???00?20??????????0-????0??10??-?0?011-00?1???00????????????????????????????????????????????????00??????200001101001---0011??0?????000?00-??000010??00100???????????010010001???11-1-1100002?1101110100??0100?0???????0010112?0??110?101001?

Erpetonyx_arsenaultorum

??1-?000000???????????0?????010----?0-??0??0???????????1?????010?2?????????10??????0????????0?????001102?0?00?1?00????0??????????0???0?????01?0???0?????????1????0?????0?11???????????1???????0??00????0?????0????????????????201001?1???????0????????1?1?0?1?110010?01?????0?002??001110010?1110????0?

Belebey_vegrandis

001-01-0000100-000000?001001000----0??0001-00-0100011011011000100010?10011?10110-1000010011001012-1--1020??00?1?0?1-??01?0000010?0?11?0-11211-2011001010110000?0???0000310---0001110101010121?100010-?????????????????????????1??????????????????????????????1???????????1???????????1?????????????????

Macroleter_poezicus

00031001000000-000001?012011011000000-0001-00-0001-2?00101100000011111101101011101000000001??0012-00-1010100(0 1)01???0???11?2000???00001?1101000200110010111120???1?1-??0???10?000101????1?1?12??00001???????????????????????????1??????1???????01???0??0????0?11???1?????????????2???????1???0?????11?0??

Milleretta_rubidgei

000210000?0100-?0100?2001001010----0??0001-00-000001?1-1011?001012100110100100-001000000101000002-0011010000000??01-??000?0111-??2?011??01?0010??10?0010112000?001-0000011010001010000?011121?0?1??0-?11?00?????????0??0??000?00100??0????00211-??00?0??100?11?0????0????1?????2???111120??001????1????

Acleistorhinus_pteroticus

000110000001?0-0?10002012101010----0??0000100-0001-0?0010??00001011011000101010001000010000101001-000101?0?0100?1?01??00???11????00011110100010?010?001?112010?00??0?0???101?0???????01?1?1??0100??????????????????????????????????????????????????????????????????????????????????????????????????????

Colobomycter_pholeter

000111-1000100-0000112111101011110000-0000000-0011-10(0 1)-1011000110110?1001101011001000010100?0?001-00-11--??0001??????????2011???0??0111101000100011?1010??20?300???00000?10??0011000101???1??0?0000????????????????????????????????????????????????????????????????????????????????????????????????????

Nyctiphruretus_acudens

000301-0000000-000000?012001020----0??0001-00-0001-1?1-101100000000010101-0100-0-10011-000-100012-00?10101?010100????????2000??002001?1101000110010?001?1120?3?1???20000111000????0000101??2??100?10-?????????1???????????????????0??1????????0??????0??1?0?0????0??0??????????20????1?2???101???00?0??

Emeroleter_levis

00031000000000-000000?012011020----0??0000200-1001-1000101100010010011101-000101-100000000-?0?010-00?101???0??1??????????????????0001???01?0020??1??101111?????1?????????10??0??????????1?????0???1????0?????0?00????1????00??1?100??1????????????1??0??11??1???00??1?00?1??????????111??00011???10011?

Deltavjatia_rossicus

100311-0011100-000010?022011000----00-0001-00-1100010000011000--010111101-000111-100001100-100010-00110101001011?001??11020001110000110-010102101?0010101121130101-10?110101000111110100011213000010-0?1?0?0???00?0?0?1?0-00??1?101101???00?20??0?11?01?1?0?0?0001??0?0??1????????0000?0?10001???1??01?

Procolophon_trigoniceps

001-01-000011110000003001001000----00-0011-00-0011-1000101100000021010?01-0010-1-10001-110-000010-00-11--101---1000-??00020?01-0?200111101001-100100001011211301?1-100130110?0011101001011121?100010-??00001?0100010?3??0-100?1?100?01???001201-0?01001?1002010001?10?00111001020000011001??0111?11?0??

Candelaria_barbouri

101-0000000000-000000?02200101100000??00?1-00-0001-0?0011?????1012001110010100-0010011-0000?0?010-00?1???????????????????2?????????011??01????1?0?????????20???0?????1?????????????????????????????????????????????????????????????????????????????????????????????????????????????????????????????????

Owenetta_kitchingorum

001-0000000000-000000?02200101100000??0001-00-0011-10001111?0000120010000-0100-0-10011-000-000010-00-11--1000010100-??0102000??00200111101001-100100001?11200301???20113011000010001001011121?100000-??0?????0?00010??000-000?100?0101???00?201-??01?0??1?1??11?00???????1?????00???11???0?????????????

Ianthasaurus_hardestiorum

0???11-1?00??0-???????01100-0311(0 1)0(0 1)0??0001-00-0?0000001100-00?111100001?110100-0110010-0110?0?002-01?10?0????????????????0??????????1???????000???01???????11?00???00?01?????0????101?0???1??1???00??1?0??22?0????0?11022-210020000??0?????????????1????10000?????????????0????101?0??????????1????????

Edaphosaurus_boanerges

011-11-1100000-000000301100-010----00-0011-00-000001?0110100001101000010110110-0110010-01100001020010103001?10001100??001000001000001?0-012?0010000110010-0110000011001101000001111110000011011??000-1????20?0?0??1?10122-2100010101?131130?1?000001000110001001020101?0110003010100000011000110100001?

Melanedaphedon_hoveneci

??1-?1??????????????????1?????0-----??0????????????????????????????????????110-0110???????????002-????????????????????????0?????????????????001????1????????????????????0101???????????????????????????????????????????????????????????????????????????????????????????????????????????????????????????

**S2.** Modified taxon-character matrix of Spindler et al. (2020) that was used in this study to explore the phylogenetic relationships of *Melanedaphodon hoveneci*.

*Archaeothyris*  00?01011??000000??0??10???????00?0???01000?0?0????????0000001?00000????00???0????0?

*Varanosaurus*

0000100011010000000101?00000?0000010?00000?0?0????????01000(0 1)0000000??1010?0?0100010

*Ophiacodon*

0000(0 1)0000101000(0 1)00010100000000000010000?00?0?0???????00(0 1)000(0 1)000(0 1)(0 1)1(0 1)0010(0 1)010?(0 1)100010

*Ianthodon* 0000000000100001?101010001001?001000??01?0?0?0????????0??0010???000??1000??????????

*Haptodus garnettensis*

0010010001000001?1111100010010(01)01001100100?0?0????????1110010?00000?000001101101101

*Palaeohatteriidae*

0010000000100001001111000100000010???00?00?0?0???????0100001010(0 1)000?10?0011011011(0 1)1

*Ianthasaurus*

100011(0 1)0??010001??1?01000110110010?1101(0 1)110101100(0 1)00011(0 1)10011?000100?(0 1)?00010101?10?

*Glaucosaurus*

?1?0(01)(01)00101?100?00001?????11??0??00????????????????????????????????????????????????

*Lupeosaurus* ?????????????????????????????????????110?101010???100000111111100?1011110?10101??1?

*E*._*novomexicanus* 111111????1?111???00?00?1111?1???????11001?10110??1?0101?1111?111111100????????1???

*E*._*boanerges* 11111111111?11101100101111110110110111100111111011010101111111111111110110111011111

*E*._*pogonias*  11111111111?1110110010111111011111?1?1100111111111011101??111?111?1101011?111?111?1

*E*._*cruciger*

11111111??1?1110??0?1?1111?1?11111???100011111111101110??11111111(0 1)11000??011???????

*Melanedaphodon*

??1?1110??1?10?0??0?????0?01??00?0?????0???????????????0???1???????????????????????

*Gordodon* 111?1110001?10?1??001001?1????1010???010010100101110011110?11???????1?????????????1
